# Human age-declined saliva metabolic markers determined by LC–MS

**DOI:** 10.1038/s41598-021-97623-7

**Published:** 2021-09-13

**Authors:** Takayuki Teruya, Haruhisa Goga, Mitsuhiro Yanagida

**Affiliations:** 1 G0 Cell Unit, Okinawa Institute of Science and Technology Graduate University, Onna-son, Okinawa, 904-0495 Japan; 2 Forensic Laboratory, Department of Criminal Investigation, Okinawa Prefectural Police HQ, Okinawa, Japan

**Keywords:** Biochemistry, Oral manifestations

## Abstract

Metabolites in human biofluids reflect individual physiological states influenced by various factors. Using liquid chromatography-mass spectrometry (LC–MS), we conducted non-targeted, non-invasive metabolomics using saliva of 27 healthy volunteers in Okinawa, comprising 13 young (30 ± 3 year) and 14 elderly (76 ± 4 year) subjects. Few studies have comprehensively identified age-dependent changes in salivary metabolites. Among 99 salivary metabolites, 21 were statistically age-related. All of the latter decline in abundance with advancing age, except ATP, which increased 1.96-fold in the elderly, possibly due to reduced ATP consumption. Fourteen age-linked and highly correlated compounds function in a metabolic network involving the pentose-phosphate pathway, glycolysis/gluconeogenesis, amino acids, and purines/pyrimidines nucleobases. The remaining seven less strongly correlated metabolites, include ATP, anti-oxidation-related glutathione disulfide, muscle-related acetyl-carnosine, *N*-methyl-histidine, creatinine, RNA-related dimethyl-xanthine and *N*-methyl-adenosine. In addition, glutamate and *N*-methyl-histidine are related to taste, so their decline suggests that the elderly lose some ability to taste. Reduced redox metabolism and muscle activity are suggested by changes in glutathione and acetyl-carnosine. These age-linked salivary metabolites together illuminate a metabolic network that reflects a decline of oral functions during human aging.

## Introduction

Metabolomics is a branch of chemical biology that profiles metabolites in cells and organisms^1,2^, using techniques such as liquid chromatography-mass spectrometry (LC–MS). It usually deals with molecules smaller than 1.5 kDa, and is an important tool for studying metabolic regulation. Using non-targeted, comprehensive LC–MS, we reported that 14 *blood* metabolites differ significantly in abundance between young and elderly people^3^. These blood metabolites may be relevant to molecular characterization of certain aspects of human aging, as they reflect changing physiological states^4,5^. We recently reported that urine contains 55 age-linked metabolites^6^. Among them, glutathione disulfide and *myo*-inositol increased exceptionally. Many declining urinary metabolites are correlated with creatinine, a waste metabolite of muscle. However, a large number of urinary metabolites are not age-related.

In this study, we report salivary metabolites that also appear linked to aging. Diverse age-linked changes occur in various tissues, so salivary metabolites may also reflect changes due to aging, and are very likely distinct from age-linked metabolites of blood and urine. Human aging appears to be an outcome with many parameters. Increased, decreased, or missing metabolites likely affect the onset and progression of aging. Comprehensive analyses regarding aging of salivary metabolites have been scarce, although age-related changes in glutathione have been reported^5,7,8^.

The current basic concept of human aging is that it constitutes a composite of processes occurring in various tissues throughout the body, at molecular, cellular, and tissue levels^9–11^. Aging events often occur in an interdependent manner so that the overall outcome of human aging may be highly coordinated. Low molecular-weight metabolites participate in numerous metabolic pathways or networks, so that their declines and increases may constitute signatures of molecular events of human aging^5^. Identified metabolites may be implicated directly or indirectly in causes of human aging.

Because saliva can be collected non-invasively, it may enable a new way to comprehensively monitor human health and disease by measuring abundances of individual salivary metabolites. For example, patients of Covid-19 lose the ability to taste and smell (used as an indicator of infection). Salivary metabolites may reflect such changes, particularly for taste changes, if salivary metabolomics of patients are thoroughly examined. We previously developed blood metabolomics to study human aging^3,12,13^. Blood metabolites may be used if precautions are taken to avoid changes in labile chemical structures. In contrast, saliva contains metabolites such as sugars, amino acids, anti-oxidants, and high-energy compounds^14–17^, some of which are important for tasting and digesting food. So far as we are aware, no comprehensive method has been established to evaluate human aging based upon abundances of salivary metabolites. Since age-related metabolites in saliva may be distinct from those of blood, they may document different aspects of human aging, enabling them to be readily diagnosed^18–23^. How they differ is of considerable interest. In urine, a number of catabolites decline in elderly samples^6^. Herein, we compare age-linked metabolic profiles of saliva with those of urine and blood.

## Results

### Sample collection and characteristics of subjects

Saliva samples were collected from 27 healthy volunteers in Onna Village, Okinawa. The average age of 14 elderly persons was 75.8 ± 3.9 year, whereas the other 13 persons were young (30.6 ± 3.2 year) (Supplementary Table S1). Their urinary metabolites had been analyzed previously^6^. The gender ratio (male/female) was 10/17 and the average BMI of subjects was 22.5 for young subjects and 24.5 for elderly. Blood glucose levels were within normal ranges (plasma glucose, 69–117 mg/dL; HbA1c, 5.1–5.8%). Two saliva samples were collected from each of the subjects at the same time. Saliva samples were quenched at – 40 °C and their extracts were stored at − 80 °C until non-targeted, comprehensive LC-MS analysis of metabolites using procedures described for blood and urinary metabolites (“Methods”)^3,6,24,25^. Procedures of LC-MS data analysis, compound identification, and statistical treatment with MZmine 2 were done as previously reported^26–28^. Replicates of all samples showed essentially identical quantification data for salivary metabolites (data not shown).

### Fourteen groups of saliva metabolites

Ninety-nine salivary metabolites, comprising 14 subgroups, were identified (Supplementary Table S2). These included 4 nucleotides, 3 nucleotide-sugar derivatives, 12 nucleosides, nucleobases, and derivatives, 4 sugar derivatives, 10 sugar phosphates, 4 vitamins and coenzymes, 5 choline and ethanolamine derivatives, 6 carnitines, 5 organic acids, 2 antioxidants, 17 standard amino acids, 10 methylated amino acids, 6 acetylated amino acids, and 11 other amino acids. Abundances of metabolites were quantified in terms of peak areas (10^6^–10^9^ arbitrary units, AU) and were also represented semi-quantitatively (low L, medium M, high H) following definitions by Chaleckis et al.^3^. Six compounds (urate, phosphocholine, arginine, phenylalanine, proline, tyrosine) were relatively abundant, ranging from H to M (H–M). Five compounds (dimethyl-xanthine, histidine, betaine, citrulline, taurine) were M (medium), while 33 additional compounds were in the range of M to L (medium to low). The remaining 55 compounds, or about half, were of low (L) abundance (Supplementary Table S3).

### Twenty-one saliva metabolites are age-linked

**Figure 1:**
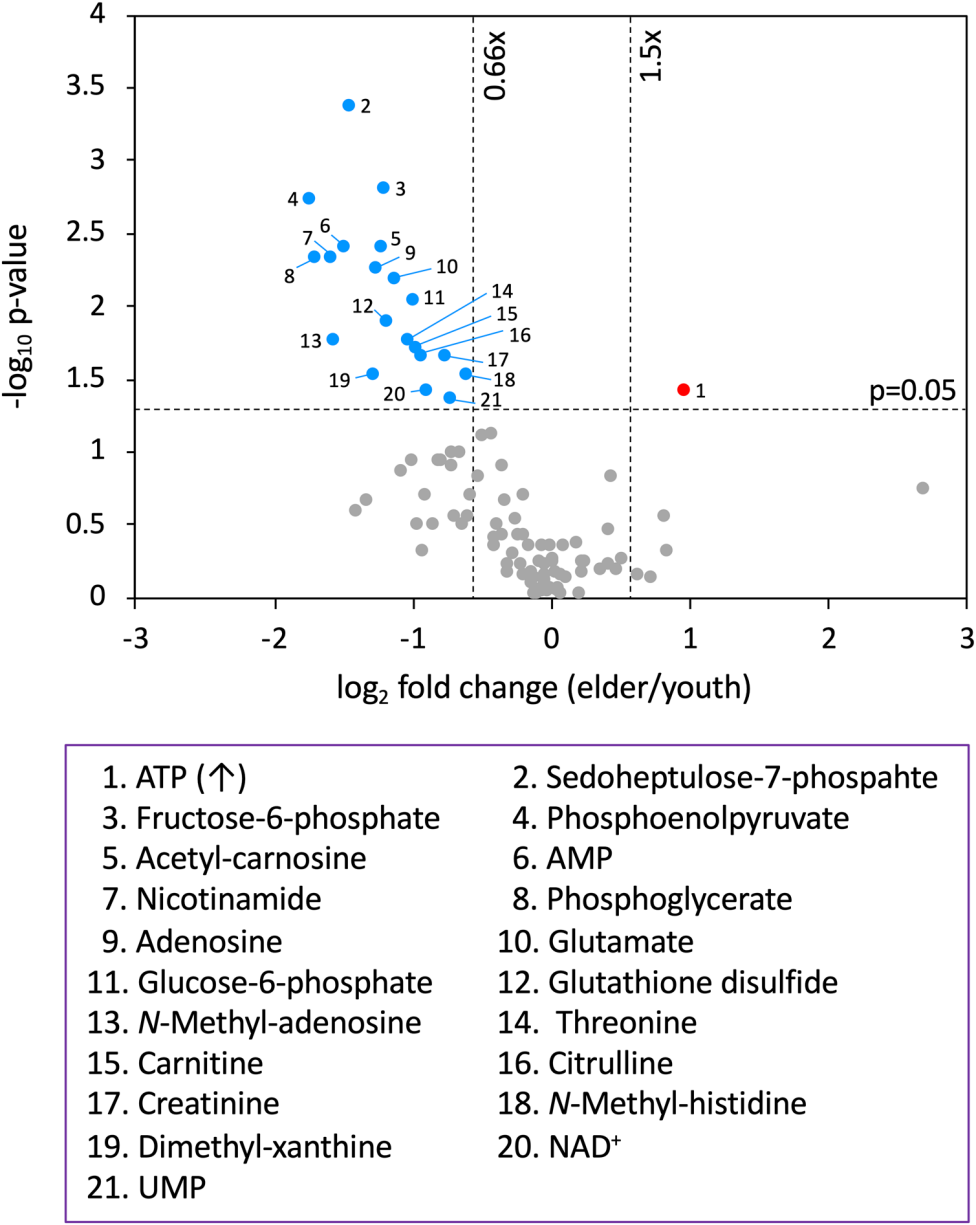
Volcano plot showing different levels of salivary metabolites in young and elderly subjects. The x-axis is the log2 fold change, whereas the y-axis is the -log10 p value of the Mann Whitney U-test. Two vertical dashed lines show the border of 0.66- and 1.5-fold change, respectively. The horizontal dashed line shows a p value = 0.05. Plots represent significantly higher (red), lower (blue), and unchanged metabolites (gray) in the elderly group. Twenty-one metabolites manifesting age differences are listed in the purple box.

Twenty-one of 99 metabolites are statistically age-related (p < 0.05, fold change < 0.66 or > 1.5) (Fig. 1, Supplementary Table S3). They comprise 3 nucleotides (ATP, AMP, UMP), 3 nucleosides (dimethyl-xanthine, adenosine, *N*-methyl-adenosine), 5 sugar phosphates, 2 vitamins/coenzymes (NAD^+^, nicotinamide), 1 carnitine, 2 standard amino acids (glutamate, threonine), 1 methylated amino acid (*N*-methyl-histidine), 1 anti-oxidant (glutathione disulfide), and 3 other amino acids (citrulline, acetyl-carnosine, creatinine).

In a volcano plot of the 99 metabolites (Fig. 1), a relatively low small change in abundance between young and elderly samples appears near the center, and metabolites that have significant p values are found on the upper side. ATP (dot no.1) was 1.96-fold more abundant in elderly subjects, yielding an elderly:young subject ratio of 1.96, whereas all other age-related compounds were less abundant in the elderly, producing ratios less than 1.0 (Fig. 1, Supplementary Fig. S1). Phosphoenolpyruvate (no. 4, ratio: 0.30), one of 5 sugar phosphates, and nicotinamide (no. 7, 0.31), a vitamin, showed the lowest ratios. In total, 7 metabolites showed peak ratios below 0.50. The majority of these belong to known metabolic pathways (KEGG, https://www.genome.jp/kegg/). In blood, 42 of these 99 compounds were enriched in RBCs (Supplementary Table S2)^3,24^, so many RBC-enriched compounds are also found in saliva. These are all sugar phosphates, nucleotide-sugars, and anti-oxidants. Metabolites of saliva thus somewhat resemble those of whole blood.

### Two salivary metabolites exhibit gender differences

We found gender differences in 2 metabolites, acetyl-carnosine and creatinine, which decrease significantly in females (Supplementary Fig. S2). Both are also linked to age (Fig. 1, Supplementary Fig. S1). Since these compounds are mainly localized in muscle, the appearance of both age and gender differences may be due to differences in oral organ or systemic muscle mass (see “Discusstion”).

### Coefficients of variation for 99 salivary metabolites among subjects

**Figure 2:**
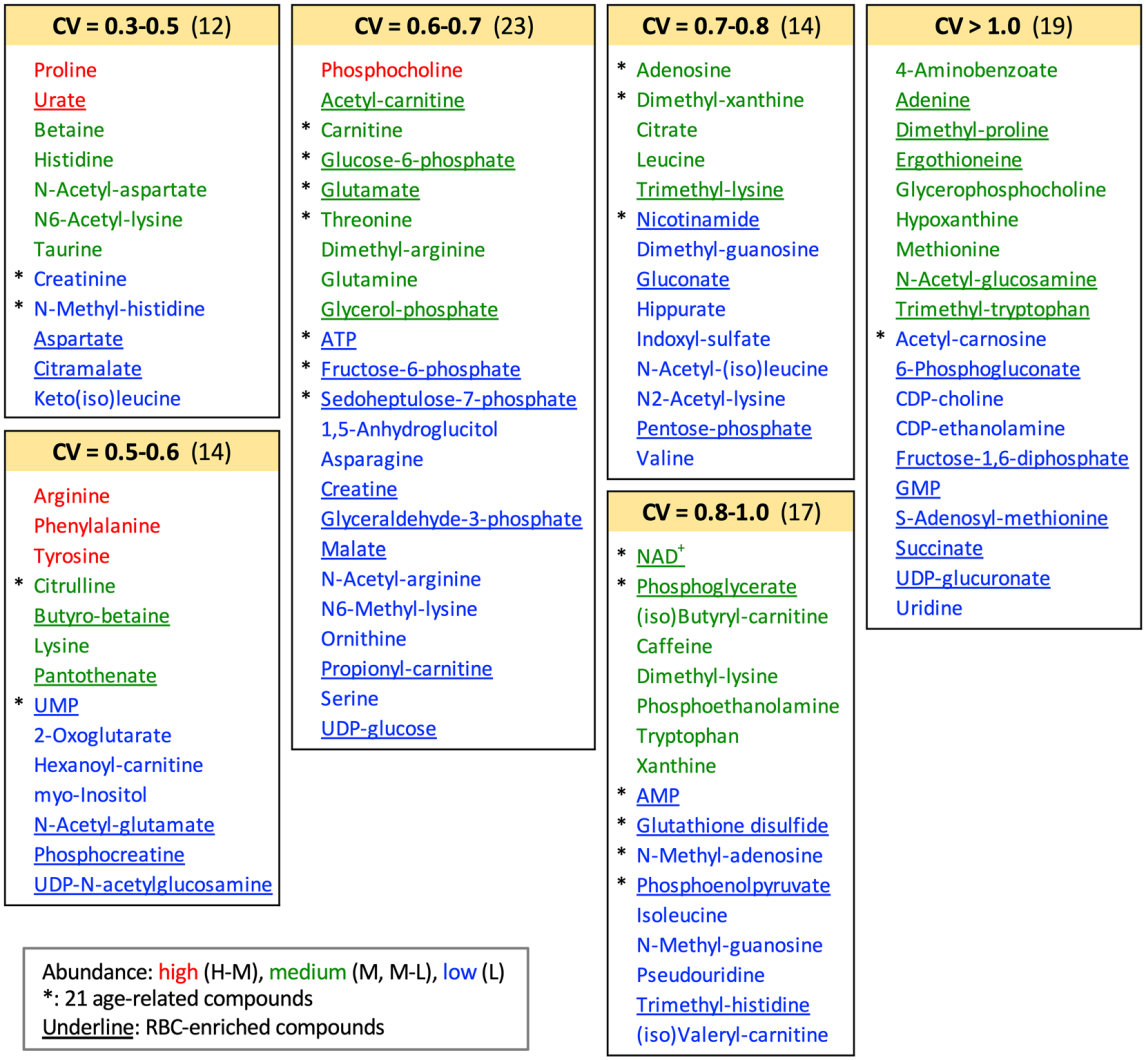
Coefficients of variation (CVs) of 99 salivary metabolites in 27 people from Onna Village, Okinawa. Compounds were classified into 6 sub-groups according to their CVs, as explained in Chaleckis et al.^3^. Numbers of compounds belonging to subgroups are shown in parentheses. Abundances of compounds are semi-quantitatively indicated by their peak areas. In blood, compounds underlined are enriched in RBCs. Compounds shown with asterisks are age-related in saliva.

Coefficients of variation (CV) represent measures of individual variability of metabolites abundances^3^. The abundance of each saliva metabolite was obtained from LC–MS data and CVs were subsequently calculated (Fig. 2, Supplementary Table S2). Salivary compounds were classified into 6 sub-groups, according to the magnitude of their CVs. Of 21 age-related salivary metabolites, only two, creatinine and *N*-methyl-histidine, belonged to the least variable group, which included 12 metabolites (CV, 0.3–0.5) (Fig. 2). Two other compounds, citrulline and UMP, were in the next least variable group, which comprised 14 compounds (0.5–0.6). Sixteen metabolites were relatively more variable, with CVs ranging from 0.60 to 1.0. Only one, acetyl-carnosine, was in the most variable (CV > 1.0) subgroup, which comprised 19 compounds. Thus, CVs of the great majority of salivary compounds (17/21) were relatively variable (CV 0.6–1.0).

### Six metabolites allow subjects to be grouped by PCA

**Figure 3:**
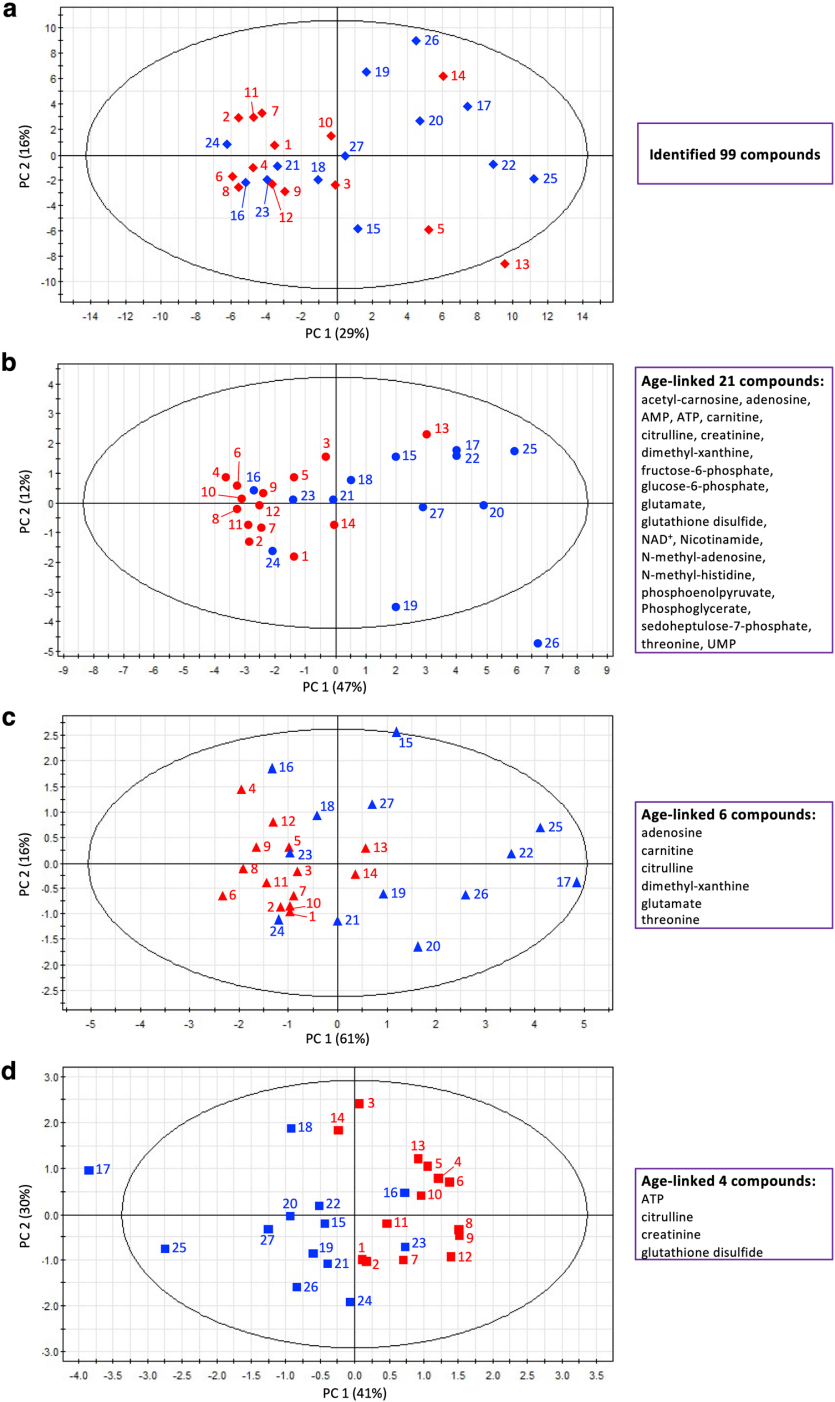
PCA of age-linked salivary compounds. (**a**) Taken together, all 99 identified metabolites cannot separate young (blue) and elderly subjects (red). (**b**) Oral aging may be detected by PCA of 21 age-linked metaoblites in a 2D manner (see text for explanation). Elderly subjects (no. 1–12, 14) are located in the negative domain of the x-axis, while young subjects (no. 15, 17–20, 22, 25–27) are located in the positive domain. However, four young subjects 16, 21, 23 and 24, appear in the negative domain, while elderly subject 13 is located in the positive domain. These positions parallel heatmap data (Fig. 4) which show metabolite levels for individual subjects. (**c**) Six metabolites can separate young and elderly subjects. (**d**) Even four metabolites largely separate them.

We employed principal component analysis (PCA) to reduce the dimensionality of abundance data for salivary metabolites. A PCA plot using all 99 compounds failed to distinguish between 13 young and 14 elderly subjects (Fig. 3a). In contrast, PCA of 21 age-linked metabolites largely separated these subjects into two groups, with elderly subjects located in the left domain, and young subjects in the right domain (Fig. 3b). The separation was imperfect, however, due to outlier subjects in whom aging appears either premature or delayed. Two of three atypical young subjects in the left domain (no. 16, 23, 24) were female and one older subject (no. 13, 80 year) in the right domain, who was exceptionally young, suggest that female subjects may be inherently more variable, based upon this limited sample.

We then attempted to reduce the number of metabolites needed to resolve young and elderly subjects. After many attempts, we found that combinations of six or even just four compounds were able to separate the subjects into two rough groups. The six compounds included adenosine, carnitine, citrulline, dimethyl-xanthine, glutamate, threonine, principally amino acids, and nucleosides (Fig. 3c), while the four were creatinine, citrulline, glutathione, and ATP (Fig. 3d).

### Correlations of age-related salivary metabolites

To further explore relationships among the 21 age-related salivary metabolites, we then derived pairwise Pearson’s correlation coefficients (r), using metabolite abundance data. Twenty-two pairs of 14 age-linked compounds showed significant (> 0.70) correlations in healthy young and elderly subjects (Supplementary Figs. S3, S4). For example, adenosine and NAD^+^-related nicotinamide had r = 0.90. Sedohetulose-7-phosphate and fructose-6-phosphate or glucose-6-phosphate, members of the pentose phosphate pathway, were highly correlated (0.85 and 0.92, respectively). Furthermore, high correlation coefficients for phosphoenolpyruvate, phosphoglycerate, and NAD^+^ (0.94) probably reflect their close association in gluconeogenesis, synthesizing glucose from smaller metabolites. This strongly suggests that specific metabolic pathways are active in saliva. In addition, carnitine, citrulline, glutamate, and citrulline are also correlated (> 0.70), suggesting that amino acid metabolism involving carnitine (muscle-related) is active.

When Pearson’s correlation analysis was extended to all 21 age-related metabolites (Supplementary Fig. S3b), 3 compounds, ATP, acetyl-carnosine, and dimethyl-xanthine were not strongly correlated (> 0.5) with any other compound, so they seemed to be more independent metabolites. At present, we are unable to offer an explanation for the lack of correlation of these compounds.

As elderly saliva contained reduced levels of metabolites related to gluconeogenesis, glycolysis, the pentose phosphate pathway, and nitrogen metabolism, it is apparent that these metabolic pathways are less active in the mouths of elderly people. ATP was the sole exception, as it increased in abundance in saliva of elderly subjects. In addition, certain nucleosides/nucleotides, such as adenosine, nicotinamide, AMP, and UMP, are correlated, and they also diminished with age so that nucleoside/nucleotide metabolism seems to be less active.

Age-dependent salivary metabolites thus reflect nutrition (carbohydrate, amino acid, nucleoside, lipid), phosphate metabolism, energy demands, redox reactions, and anti-oxidation. These are consistent with the presence of ATP, NAD^+^, glutathione disulfide, carnitine, and creatinine. Notably, these age-related salivary metabolites lessen with age, consistent with the notion that aging in saliva might be caused by the loss of some essential metabolic (digestion, energy, muscular) features of oral activities. The lone exception is the increase of ATP. The reason for this increase is unclear, but possibly it is due to reduced catabolism of ATP to ADP and AMP.

### Heatmap of metabolites reveals the mode of oral aging

**Figure 4:**
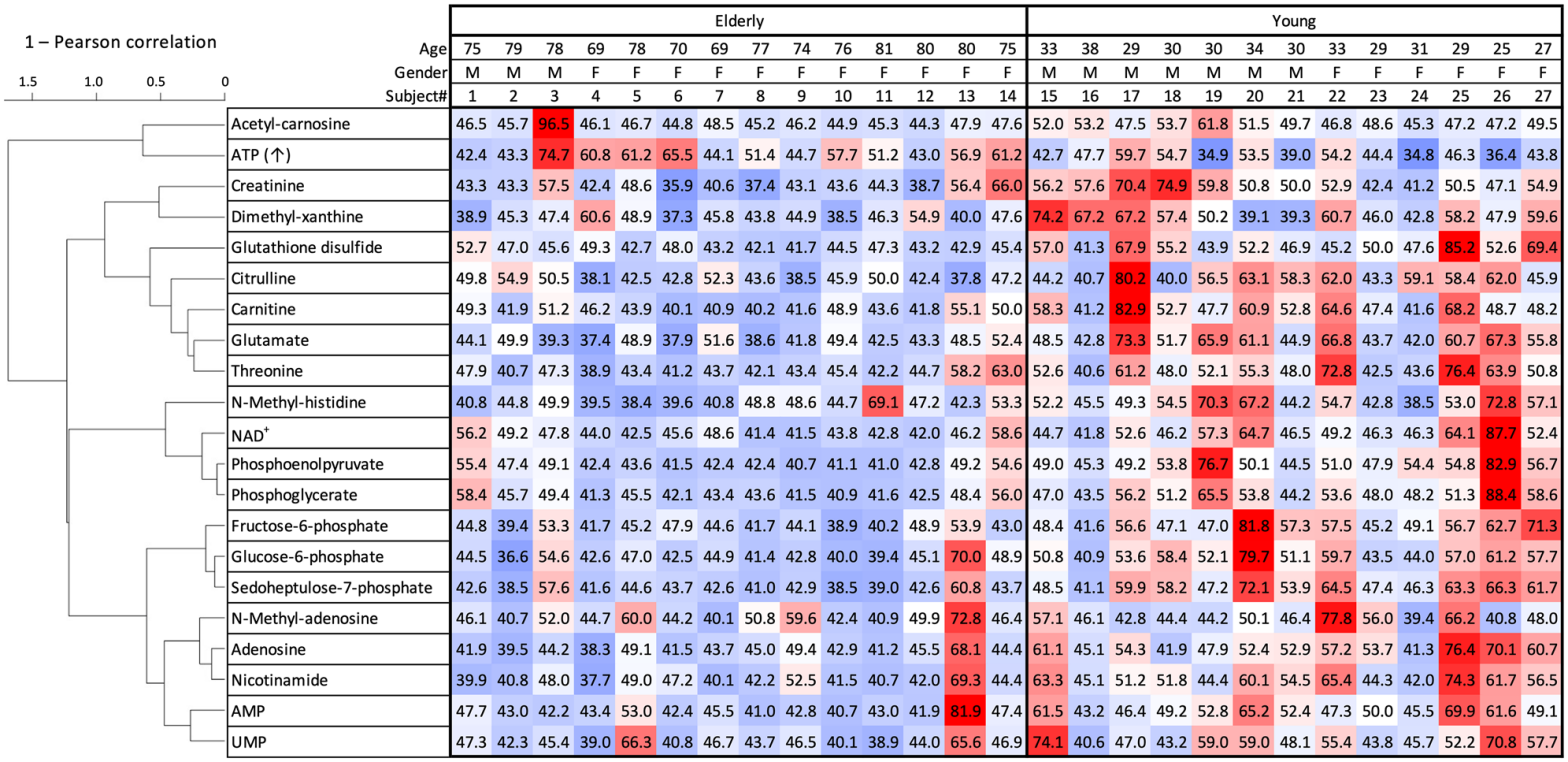
Hierarchical clustering heatmap of 21 salivary age-related metabolites in 14 elderly and 13 young subjects. Correlations between compounds are reflected by bar lengths, which is consistent with the correlation network data (Supplementary Fig. S3). Standardized scores (T scores) for each metabolite are represented by colors. The average value (50), white; values above average, red; values below average, blue. Color intensity of the cells reflects the T score. The cluster dendrogram was created by using R. Microsoft Excel was used to calculate T scores and create the heatmap.

To estimate the degree of salivary aging among individuals, a heatmap approach was taken**.** Abundances of 21 age-linked salivary metabolites were colored, reflecting the degree of deviation from average, with blue representing abundances below average, and red, higher than average (Fig. 4). Young subjects clearly display higher levels of age-related metabolites (subjects 15–27, red) and senior subjects (1–14) show decreased metabolites (blue).

Two elderly female subjects (13 and 14, 80 and 75 years, respectively) revealed surprisingly young patterns, particularly no. 13. Scrutinizing the heatmap of no. 13, rather high levels of energy-related metabolites (*N*-methyl-adenosine, adenosine, AMP, UMP, sedoheptulose-7-phosphates, glucose-6-phosphate, and fructose-6-phosphates), and redox and muscle metabolites (nicotinamide, carnitine, and creatinine) are evident. This level of nucleotide-related metabolism may be exceptional.

## Discussion

In this study, comprehensive quantification of human salivary metabolites was conducted using LC–MS. Among 99 metabolites, 21 proved to be age-related. All but one of these age-linked metabolites declined with age, and only ATP increased in the elderly. The degree of age-related decline ranged from 30% for phosphoenolpyruvate to 65% for *N*-methyl-histidine. These metabolites were reproducibly quantified in duplicate. Our results indicate that investigations of human aging focused on individual diversity in saliva metabolite levels may be revealing. For this purpose, improved sample storage method and simple, and rapid analysis should be established in the future.

About half of age-linked saliva metabolites were highly correlated (Supplementary Fig. S3), forming a correlation network (Supplementary Fig. S4). Five sugar phosphates suggest that the pentose phosphate and glycolysis/gluconeogenesis pathways are active in saliva and that their activities decline in elderly people. Other age-linked metabolites such as NAD^+^, nicotinamide, adenosine and AMP also declined in elderly subjects, supporting the notion that antioxidation and energy are important for salivary metabolism and diminish in elderly subjects. To our knowledge, these metabolic pathways in saliva have not been recognized previously.

Amino acids (glutamate, threonine, citrulline, carnitine) are also age-related. Glutamate is the major excitatory neurotransmitter in the brain, involved in functions such as motor behavior, cognition, and emotion. It may be affected in the course of normal aging. Glutamate concentration decreases with age predominantly in the gray matter motor cortex region^29^. Threonine produces glycine in the process of catabolism to pyruvate and is used to in the to synthesize collagen, glutathione, creatine, and so on. Citrulline is a by-product of nitric oxide (NO) synthesis in the urea cycle, and a citrulline deficiency can cause a reduction in the bioavailability of NO. Carnitine is related to mitochondrial function in muscle and brain^30–33^. Hence oral functions related to these metabolites may decline in the elderly. Comprehensive quantitative analyses of salivary metabolites are scarce so many of age-linked metabolites have not been reported in the literature (see Nassar et al.^7^).

Metabolites such as ATP, creatinine, acetyl-carnosine, glutathione disulfide, and methyl-histidine do not participate in a clear correlation network (r < 0.7), but depict salivary aging demonstrated by their decline or increase (ATP) (Fig. 1). We confirmed a previous report on the decline of glutathione disulfide^7^. Not only glutathione, but other anti-oxidative compounds are quite abundant in saliva, such as anserine, which contains *N*-methyl-histidine, acetyl-carnosine. Acetyl-carnosine, creatinine, and *N*-methyl-histidine are also authentic muscle-related metabolites, perhaps required to support tasting and other lingual and oral activities. The reduction of these three muscle specific metabolites in saliva is symbolic among the aging markers identified in this study. *N*-Methyl-histidine is a component of the muscle dipeptide, anserine. *N*-Methyl-histidine concentration in saliva increases after exercise^34^, as well as in blood and urine^35^. We recently reported that the urinary *N*-methyl-histidine level in elderly people was significantly lower than in young people^6^. Thus, a decrease in salivary *N*-methyl-histidine in the elderly may reflect a decrease in systemic basal muscle mass and physical activity with age. Interestingly, acetyl-carnosine and creatinine also showed a gender difference (Supplementary Fig. S2). Acetyl-carnosine with antioxidant activity is mainly localized in muscles and has a role in suppressing inflammation or fatigue^36^. Non-invasive measurement of free carnosine in muscle by proton magnetic resonance spectroscopy (1H-MRS) showed that carnosine content increased dramatically in puberty (8–20 year) boys, but not in girls. In addition, a decrease was observed from young adults (21–30 year) to adults (31–50 year) for both men and women, but there was no significant change in adults and elderly (60–83 year)^37^. Creatinine is a metabolite of creatine phosphate, which is a source of muscle energy, and its serum and urinary concentrations are highly correlated with skeletal muscle mass^38,39^. Hence, the age and gender differences of these two muscle metabolites in saliva may be closely related to differences in oral organ or systemic muscle mass. In addition, amino acids or peptides, such as anserine and glutamate are involved in taste, so their decline suggests that elderly people lose their ability to taste. Some of these age-linked compounds (14/21) formed a clear high correlation network, but others did not (Supplementary Fig. S4). When PCA was tested in more than 20 combinations from the 21 compounds, we found that even as few as 4 compounds, ATP, citrulline, creatinine, and glutathione disulfide, roughly discriminate age (Fig. 3d). Judging from the diversity of age-linked salivary metabolites, numerous physiological mechanisms are reflected in salivary aging and appear coordinated. Anti-oxidative, redox, and energy production diminish in elderly subjects.

**Figure 5:**
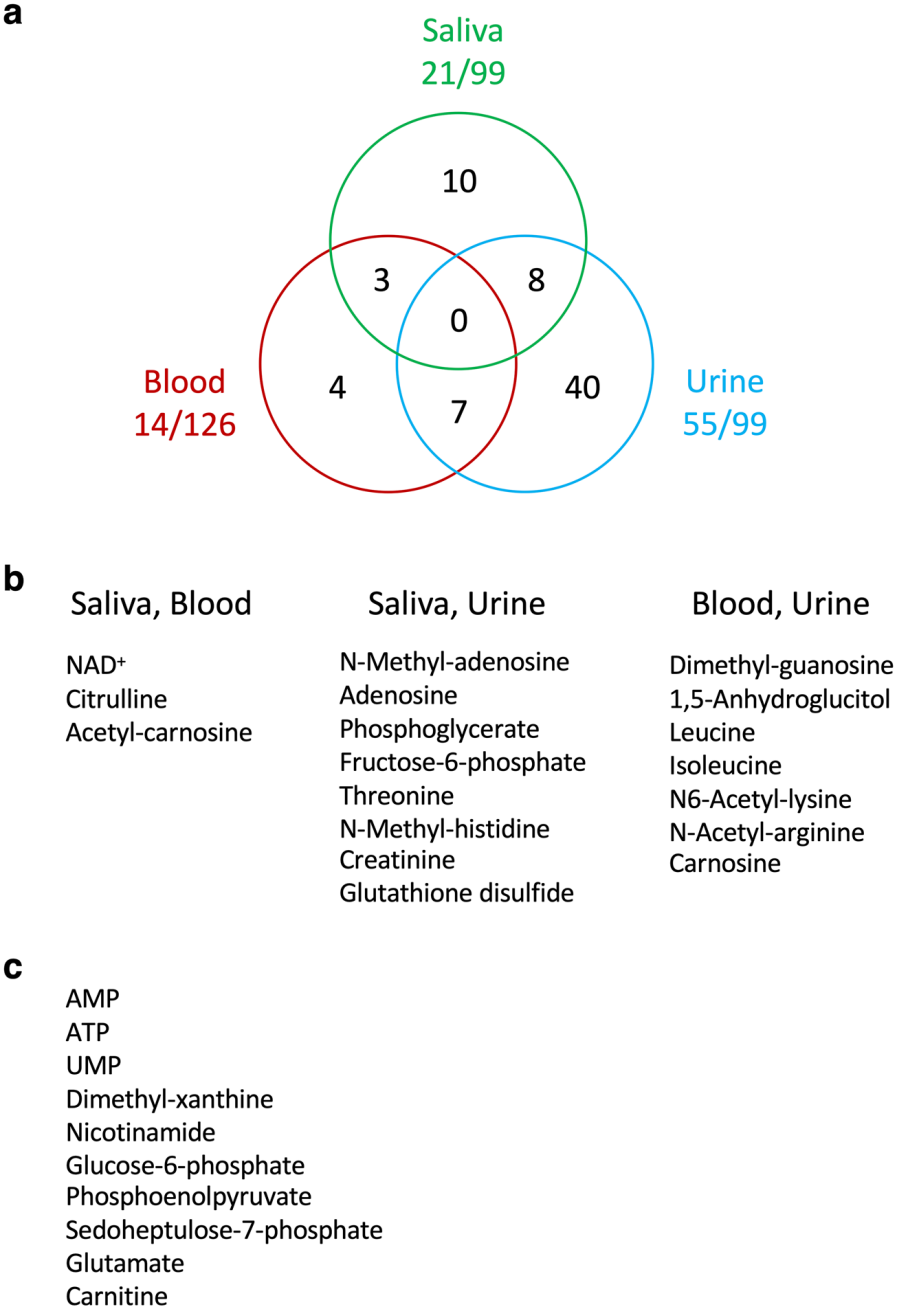
Common human age-related metabolites in saliva, blood, and/or urine. (**a**) Numbers of shared compounds in overlapping regions. No compound was common to saliva, blood, and urine. Three, 7, and 8 compounds were common between saliva and blood, between blood and urine, and between saliva and urine, respectively. (**b**) Three, 8 and 7 age-linked compounds were found commonly between saliva and blood, between saliva and urine, and between blood and urine, respectively. (**c**) Ten salivary age-linked metabolites that did not overlap with blood or urine age-linked metabolites.

We previously reported 14 and 55 age-related metabolites in blood and urine, respectively^3,6^. These studies employed rapid quenching of samples developed in this laboratory. In this study, we compared these two groups of metabolites with 21 age-linked salivary metabolites. No single age-linked metabolite was common to blood, urine and saliva, consistent with the physiologically distinct functions served by the tissues that form them (Fig. 5a). However, 3, 8 and 7 metabolites, were common to blood and saliva, saliva and urine, and blood and urine, respectively (Fig. 5a,b). Thus NAD^+^, citrulline, and acetyl-carnosine are age markers common to blood and saliva. Their decline may represent decreased redox and muscular performance supported by these metabolites in blood and saliva.

On the other hand, 8 metabolites were age markers shared between urine and saliva (Fig. 5b). Carbohydrate metabolites, phosphoglycerate and fructose-6-phosphate, are implicated in glycolysis/gluconeogenesis and the pentose phosphate pathway, respectively. Muscle-related *N*-containing creatinine and *N*-methyl-histidine, and anti-oxidant glutathione disulfide were present in both saliva and urine. Creatinine is a catabolite of muscle metabolism, and a well-known age marker in urine^40,41^. *N*-Methyl-histidine is a degradation product of the muscle-activating dipeptide, anserine^42,43^. While urine and saliva share these age-linked metabolites, they may be present for excretion or degradation in urine, but in saliva they may function in nutrition rather than excretion.

Between urine and blood, seven common metabolites were found. Three of them, dimethyl-guanosine, *N*6-acetyl-lysine and *N*-acetyl-arginine, are uremic toxins^44–48^ when they are abundant. Indeed they increase in elderly blood, but decrease in urine of healthy elderly persons, suggesting that they do not cause kidney impairment in healthy people. Consequently, these three uremic toxins were not detected in saliva in any of our samples. Leucine, isoleucine, muscle-related carnosine, and 1,5-anhydroglucitol (1,5-AG) decline in elderly people. The level of 1,5-AG decreases if blood glucose increases, as it competes with glucose for reabsorption^49^. Thus, age-linked metabolites overlap partially in blood, urine, and saliva. In addition, ten salivary metabolites are specifically age-linked (Fig. 5c). Three are phosphorylated nucleotides, and three are sugar phosphates. The remaining four compounds are nitrogen-containing amino acids and a base, which may act as anti-oxidants and may be related to vitamins and to caffeine. Roles of saliva-specific, age-linked compounds may be worthy of investigation.

Mechanisms that explain how common metabolites in saliva, urine, and blood decline in parallel during aging are little known. Future studies of salivary aging may reveal age-linked oral decay leading to diseases, such as cancers of the tongue or secretory organs^50–53^. Since the composition of age-linked metabolites in saliva is partly identical to those of blood and urine, certain aging mechanisms may also be shared. Since NAD^+^, citrulline, and acetyl-carnosine are common to both saliva and blood, the decay in redox maintenance, regulation of ammonium and anti-oxidation may involve certain cross talk mechanisms.

Strikingly, many metabolites that were not affected by aging are uncorrelated, indicating that highly correlated metabolites may be affected by oral aging. This is the subject of a future study. Thus, in saliva, age-linked compounds are related to relatively broad metabolic conditions so that age-related information obtained from salivary metabolites may also be distinct from that of blood and urine. However, correlation coefficients of these metabolites are rather high, so aging processes may be highly interrelated so that they can be simplified and/or unified. Saliva may greatly help to assess the degree of human metabolic aging. Salivary metabolites may also prove useful to better understand pediatric matters. Human characteristics monitored via salivary compounds undoubtedly have broad significance.

## Methods

### Sample collection and preparation

Twenty-seven healthy male and female volunteers participated in this study. Saliva samples were taken in the morning and subjects were asked not to eat breakfast to ensure at least 8 h of fasting. During fasting, they took water freely. After rinsing the mouth lightly with water, ~ 1 mL saliva samples for metabolomic analysis were spit into 50 mL plastic tubes. It is suggested that saliva collection without stimulation at rest, such as in the morning, is important to ensure reproducibility in quantification of saliva metabolites^54,55^. Saliva samples were collected at the laboratory within 3 h. 0.2 mL saliva were quenched in 1.8 mL of 55% methanol at − 40 °C. Metabolomic samples were prepared as reported previously^3^. Briefly, two internal standards (10 nmol of HEPES and PIPES) were added to each sample. After brief vortexing, samples were transferred to Amicon Ultra 10 kDa cut-off filters (Millipore, Billerica, MA, USA) to remove proteins and cellular debris. After sample concentration by vacuum evaporation, each sample was re-suspended in 40 μL of 50% acetonitrile, and 1 μL was used for each injection into the LC–MS system.

### LC–MS analysis and data processing

LC–MS data were acquired using an Ultimate 3000 DGP-3600RS HPLC system (Thermo Fisher Scientific, Waltham, MA, USA) coupled to an LTQ Orbitrap mass spectrometer (Thermo Fisher Scientific, Waltham, MA, USA)^3,12^. Briefly, LC separation was performed on a ZIC-pHILIC column (Merck SeQuant, Umea, Sweden; 150 mm × 2.1 mm, 5 μm particle size). Acetonitrile (A) and 10 mM ammonium carbonate buffer, pH 9.3 (B) were used as the mobile phase, with a linear gradient from 80–20% A in 30 min at a flow rate of 100 μL/min. The mass spectrometer was operated in full-scan mode with a 100–1000 m/z scan rate and automatic data-dependent MS/MS fragmentation scans. For each metabolite, we chose a singly charged, [M+H]^+^ or [M−H]^−^, peak (Supplementary Table S2). Peak detection and identification of metabolites were performed using MZmine 2 software (http://mzmine.github.io/)^26^. Detailed data analytical procedures and parameters have been described previously^25^. Metabolite peaks were identified by comparing their m/z values and retention times with pure standards listed in previous reports^3,24,25,56^.

### Statistical analysis

Peak data processed with MZmine 2 were exported into spreadsheet format and analyzed with R version 3.2.1 statistical environment (http://www.r-project.org). Statistical analysis was performed using the Mann Whitney U-test, with statistical significance assumed at p < 0.05. Pearson’s correlation coefficient was calculated using Microsoft Excel. The score plot of principal components (PC) was generated with SIMCA-P+ software (Umetrics Inc., Umea, Sweden). The synthetic degree of aging is expressed in two dimensions (2D) with the first principal component representing mostly aging, while the second component quantifies additional aging dependent on metabolites. A heatmap represents standardized abundance data for each metabolite in each individual. Color intensity of cells reflects t-scores, indicating levels higher or lower than average (50). T-scores were calculated from the following formula: T-score = [(sample peak area − average of population peak area) × 10/standard deviation of population peak area] + 50. Thus, the mean and standard deviation are 50 and 10, respectively.

### Ethical approval

Written, informed consent was obtained from all donors, in accordance with the Declaration of Helsinki. Experiments were performed in compliance with relevant Japanese laws and institutional guidelines, and protocols were approved by the Human Subjects Research Review Committee of the Okinawa Institute of Science and Technology Graduate University (OIST).

## Supplementary Information

Supplementary Information 1.

## Data Availability

Raw LC–MS data in mzML format are accessible via the MetaboLights repository (http://www.ebi.ac.uk/metabolights). Data for the 27 volunteers are available under accession number MTBLS2108.
