# Supplementary material for: Human age-declined saliva metabolic markers determined by LC–MS
Source: Sci Rep. 2021 Sep 13;11:18135. doi: 10.1038/s41598-021-97623-7 (PMC8437986; doi:10.1038/s41598-021-97623-7)
Supplement: Supplementary file 1 — Supplementary Information 1. [file 41598_2021_97623_MOESM1_ESM.docx]

**Supplementary Information**

**Human age-declined saliva metabolic markers determined by LC-MS**

**Takayuki Teruya^1^, Haruhisa Goga^1,2^ and Mitsuhiro Yanagida^1*^**

^1^G0 Cell Unit, Okinawa Institute of Science and Technology Graduate University, Okinawa, Japan

^2^Forensic Laboratory, Department of Criminal Investigation, Okinawa Prefectural Police HQ, Okinawa, Japan (on leave of absence)

*****Correspondence: Mitsuhiro Yanagida (myanagid@gmail.com)

This file includes Supplementary Tables S1-S3 and Figures S1-S4.

**Supplementary Table S1.** Collection of saliva samples. Samples were collected from 27 subjects in Onna Village, Okinawa. The average age, gender, BMI, and glycemic parameters in blood are shown.

|  | Youth (n=13) | Elderly (n=14) | All (n=27) |
| --- | --- | --- | --- |
| Age | 30.6±3.2 | 75.8±3.9 | 54.0±22.9 |
| Gender (male/female) | 7/6 | 3/11 | 10/17 |
| BMI, kg/m^2^ | 22.5±2.7 | 24.5±3.7 | 23.5±3.4 |
| Plasma glucose, mg/dL | 82.2±5.7 | 91.3±10.7 | 86.9±9.8 |
| Hemoglobin A1c, % | 5.1±0.2 | 5.4±0.2 | 5.3±0.3 |

**Supplementary Table S2.** Ninety-nine metabolites identified in saliva, with coefficients of variation among 27 subjects.

**Supplementary Table S2.** (continued)

**Supplementary Table S2.** (continued)

**Supplementary Table S3.** Ninety-nine salivary metabolites identified in the present study. Abundances of 99 saliva metabolites are shown (high H, medium M and low L, determined from peak areas in mass spectrometry). P-values between age or gender groups were calculated with the Mann Whitney U-test. Twenty-one metabolites linked to aging are indicated by asterisks.

 **Supplementary Table S3.** (continued)

 **Supplementary Table S3.** (continued)

**Supplementary Figure S1.** Dot plot profiles of 21 salivary aging markers for 27 subjects (14 elderly and 13 young). Pale red and azure dots represent elderly and young subjects, respectively. Bars represent medians in each group. Peak ratios of median values (elderly/young) are shown in parentheses.

**Supplementary Figure S2.** Acetyl-carnitine and creatinine showed gender differences. (**a**) Volcano plot for different levels of salivary metabolites in females and males. The x-axis is the log2 fold change, whereas the y-axis is the -log10 p-value of the Mann Whitney U-test. Two vertical dashed lines indicate 0.66 and 1.5 fold change, respectively. The horizontal dashed line indicates p > 0.05. (**b**) Dot plot profiles of acetyl-carnosine and creatinine for 27 subjects. Bars represent medians in each group. Peak ratios of median values (female/male) are shown in parentheses.

**Supplementary Figure S3.** Correlation among age-linked salivary metabolites revealed by Pearson’s correlation analysis. (**a**) Correlation coefficients (r) plotted for 11 salivary aging markers for 27 subjects. Abbreviations: Thr, threonine; S-7-P, sedoheptulose-7-phosphate; G-6-P, glucose-6-phosphate; F-6-P, fructose-6-phosphate; Glu, glutamate; PE, phosphoenolpyruvate; PG, phosphoglycerate. When correlation coefficients were close to 1.0, they were plotted along the diagonal line (e.g., r = 0.92 between sedoheptulose-7-phosphate and glucose-6-phosphate). When r was low, as between adenosine and citrulline (0.26), r values were randomly located. (**b**) Correlation map of 21 salivary age markers. Red unit correlation > 0.7, yellow unit correlation 0.5-0.7. The highest correlation was 0.94, between phosphoenolpyruvate and phosphoglycerate. Three metabolites, acetyl-carnosine, ATP and dimethyl-xanthine, did not show significant correlations to other age-linked metabolites (see text).

**Supplementary Figure S4.** A correlation network formed by age-related compounds in saliva. Fourteen age-linked salivary metabolites form a high correlation network (r > 0.7), involving amino acid metabolism, glycolysis/gluconeogenesis, the pentose phosphate pathway (PPP), and nucleobase metabolism, whereas 7 other salivary metabolites surrounded by broken lines are less well correlated. Three compounds, ATP, dimethylxanthine, and acetyl-carnosine were least correlated (see Supplementary Fig. 3b).
